# Barriers and drivers of public engagement in palliative care, Scoping review

**DOI:** 10.1186/s12904-024-01424-4

**Published:** 2024-05-07

**Authors:** Pilar Barnestein-Fonseca, Alicia Nebro-Gil, Virginia P. Aguiar-Leiva, Eva Víbora-Martín, Inmaculada Ruiz-Torreras, Maria Luisa Martín-Rosello, on behalf iLIVE Group, Agnes van der Heide, Vilma Tripodoro, Verónica I. Veloso, Silvina Montilla, Gustavo G. De Simone, Gabriel Goldraij, Mark Boughey, Michael Berger, Claudia Fischer, Judit Simon, Raymond Voltz, Melanie Joshi, Julia Strupp, Svandis Iris Halfdanardottir, Valgerdur Sigurdardottir, Berivan Yildiz, Ida J. Korfage, Anne Goossensen, C. van Zuylen, Eric C. T. Geijteman, Simon Allan, Dagny Faksvåg Haugen, Grethe Skorpen Iversen, Urska Lunder, Misa Bakan, Hana Kodba-Ceh, Carl Johan Fürst, Maria E. C. Schelin, Steffen Eychmüller, Sofia C. Zambrano, John Ellershaw, Stephen Mason, Tamsin McGlinchey, Ruthmarijke Smeding

**Affiliations:** 1 Instituto de Formación E Investigación CUDECA, Fundación CUDECA Instituto IBIMA-BIONAND, Grupo CA15 Málaga, Spain; 2 iLIVE Consortium , https://www.iliveproject.eu/

**Keywords:** Barriers and Facilitators/drivers, Community engagement, Palliative Care, Public engagement

## Abstract

**Background:**

The integral model of Palliative Care recognizes the community as essential element in improving quality of life of patients and families. It is necessary to find a formula that allows the community to have a voice. The aim of this scoping review is to identify barriers and facilitators to engage community in PC.

**Methods:**

Systematic search was conducted in NICE, Cochrane Library, Health Evidence, CINAHL and PubMed database. Keywords: Palliative care, End of life care, community networks, community engagement, public engagement, community participation, social participation, barriers and facilitators.

**Results:**

Nine hundred seventy-one results were obtained. Search strategy and inclusion criteria yielded 13 studies that were read in detail to identify factors influencing community engagement in palliative care, categorized into: Public health and public engagement; Community attitudes towards palliative care, death and preferences at the end of life; Importance of volunteers in public engagement programs; Compassionate communities.

**Conclusion:**

Societal awareness must be a facilitated process to catalyse public engagement efforts. National policy initiatives and regional system support provide legitimacy and focus is essential for funding. The first step is to get a sense of what is important to society, bearing in mind cultural differences and to channel those aspects through health care professionals; connecting the most assistential part with community resources. The process and long-term results need to be systematically evaluated.

## Background

Society, professionals and healthcare systems are heavily focused on treating illnesses, and sometimes can lose focus on the inevitability of death [1]. Current models of end-of-life care are being questioned because of their excessive professionalization, the overload and saturation of palliative care (PC) resources in the face of growing demand. Hence the importance of seeking formulas that provide innovative and integrated responses for the need to ensure continuity of care at home, and the complexity of responding to the emotional, socio-familial and spiritual needs inherent to the dying process [1].

Caring for people at the end of life has traditionally been normal and routine for families and communities, as has the care and support of people in the bereavement process. Moreover, they have the experience and knowledge of how to provide this help, a value that is little recognized and integrated into our health care systems [2]. Over the last 100 years in Western Europe, we have witnessed a growing disconnection between the basic family unit and the more distant family and wider community networks. Palliative care programmes over the last 50 years have developed, as communities have become increasingly disconnected from the dying process [3]. The aging of the population is making caregivers older and frail with a significant degree of social isolation, while younger caregivers, even neighbours themselves, have busy lives, unable to engage in caregiving thus making caregiving a burden. This has encouraged the professionalization of caregiving [3].

The integral model of palliative care recognizes the community as an essential element in improving the quality of life of patients and their families, and it is necessary to find a formula that truly allows the community to have a voice in the design and implementation of palliative care policies and initiatives. In this way, the Health Promoting Palliative Care model [HPPC] [4] advocates for movement towards a sustainable social model of end-of-life care, where death and dying are considered within the community context of everyday life and where each social actor is empowered to contribute. A key principle of building community capacity includes normalizing death and preparing communities for end of life [5, 6].

The well-known universal strategy of “Healthy cities", a holistic concept that depends on the physical, social, political, economic and spiritual environment and not only on the quality of health care [2], must take into account that, despite their best efforts, they will have to face a certain burden of death and loss. In the last few years, the links among palliative care, social justice, and human rights have been strengthened, with international recognition right up to the level of the World Health Organization. It is recognized that the right to healthcare includes the right to accessing good quality palliative care. [7].

The current reality of loneliness and individualism in our society must make us revise the ideas and practices of "Healthy Cities" by also making them compassionate, this refers to people reconnecting with the most "human" care, getting involved and creating supportive networks in care. This concept is what is called "Third Wave Public Health" because it incorporates the experiences of death and loss in our health formulations and includes the idea of compassion in our health policies [2]. To achieve this, the key objective is to sensitize the community to re-engage in end-of-life care.

A public health approach to PC is a health promotion approach to end-of-life care, one that sees the community as an equal partner in the long and complex task of providing quality end-of-life care. Just as health, according to WHO (World Health Organization), is "everyone's responsibility," so too is death, loss, and care. A great example of this is The Compassionate Cities movement and their Compassionate Cities "Charter for Action" promoted by the PHPCI Organization [8], where they define the Compassionate cities are “those that publicly recognize people at the end of life and their needs, and are conscious of seeking and involving all major sectors of the city to help through care and accompaniment to reduce the social, psychological and health impact of difficult life processes and situations, especially those related to disability, ageing, dependency, end of life, caregiver burden, grief and bereavement”. It is the real commitment of the people and organizations working in the community who possess the skills and experiences necessary to bring about change.

However, a significant proportion of UK adults report not being comfortable discussing death and dying with family and friends. And international research shows that members of the public are rarely familiar with the meaning and availability of palliative care and that the majority have not taken steps to anticipate their own future care through the use of advance care planning [4, 6, 7].

One of the basic keys sensitizing and public engagement is to know what society is like and what it can offer, identifying and bringing together the values, resources and experience it already has, 'it’s assets'[9]. This strategy is in line with the public health approach to palliative care and catalyses community participation through four fundamental steps [10]:

1. Identification and participation of all social agents, associations and public entities. The community that wants to get involved in end-of-life care identifies its strengths and assets; but generally does not know how to get started. In these first steps health, social systems and public entities must identify their resources for the community in question, to lead and facilitate the process.
2. Meeting: To make people and organizations involved feel heard. It helps them recognize opportunities to work together in building and learning from each other's strengths
3. Action plan: It requires acknowledgement and partnerships to be established; all actors involved in end-of-life care must develop a well-structured action program that reflects the needs of the target community with clear objectives.
4. Implementation: implementation of the initiative and evaluation of the program's effectiveness.

Although these four steps seem easy and clear, their development has allowed us to detect some barriers: there is a resistance to asking for and accepting help, as well as thinking about the end of life and death as a natural part of life. Cultural differences are increasing and becoming very evident due to the rise of immigration. People lack knowledge about palliative care; their main source is based on personal experience and unfortunately, most available educational material on palliative care for the general public on Google does not meet the standard, helping common misconceptions prevail [11–13].

Public engagement is an activity that requires a focus of more than one organization and community-wide awareness requires several organizations to be involved from the beginning [10]. Making the need for health and social networks for chronically ill patients with social needs apparent is a challenge, and the identification of patients and health, social and community services involved in palliative care is often complicated [10].

The aim of this scoping review is to identify barriers and facilitators to engage community in palliative care.

## Methods

A scoping review was identified as the most appropriate method to identify barriers and facilitators to engage the community in palliative in the literature and to identify any existing gaps in knowledge, aligned with suggestions that this method of evidence synthesis is especially useful for gaining insight into programs [14, 15].

This scoping review was conducted in accordance with the Preferred Reporting of Items for Systematic Reviews and Meta-Analyses—extension for scoping reviews (PRISMAScR, see Supplementary Document S2) [16].

### Eligibility criteria

Studies were eligible for inclusion if they were peer-reviewed journal articles published between 2016 and December 2022, with no language limitation, and had to address community participation in palliative care as a main topic, as well as the difficulties and strengths for their development. The search included empirical studies (qualitative and quantitative) and systematic reviews. The methodological quality of the studies was not assessed due to the scarcity of relevant literature and the heterogeneity of the studies.

### Information sources

To identify relevant studies, a systematic search was conducted in NICE (National Institute for Health and Care Excellence), Cochrane Library, Health Evidence, CINAHL (Cumulative Index of Nursing and Allied Literature Complete) and PubMed databases.

### Search strategy

Search terms were developed, reviewed, and refined by the full research team. The following search strategies were used to elicit a broad coverage of the extant literature. This strategy was used for all data base.

- Palliative care OR end of life care AND community engagement OR public engagement OR community participation AND barriers and facilitators
- “Palliative care” [MESH] AND “Community Networks” [MESH]
- “Palliative care” [MESH] AND “Community networks” [MESH] AND Barriers and facilitators.
- Palliative care OR end of life care AND community engagement OR public engagement OR community participation
- Palliative care OR end of life care AND community engagement OR public engagement OR community participation AND barriers and facilitators
- Barriers and facilitators AND social implications AND palliative care
- Social implications AND palliative care

### Selection of sources of evidence

Using the above search terms and strategies, and after eliminating duplicates, the two lead authors assessed titles, keywords and abstracts. Full texts of studies that met the inclusion criteria, including those whose relevance was unclear, were obtained and reviewed. Two authors reviewed each of the full texts against the inclusion criteria. This independent review resulted in 96% agreement among the reviewers. A third reviewer resolved disagreements, so that all three reviewers agreed on the final criteria for article selection.

### Data charting & data items

Data recording and extraction was conducted by the principal investigator using an iterative process, in consultation with the research team. Data from eligible studies were recorded using a standardised data extraction model designed for this study, relevant information on key study characteristics and detailed information was extracted. The following characteristics were extracted: article characteristics (author, year of publication, country); focus (community impact, survey and community intervention); target population (health professionals, patients, carers, volunteers); key findings related to barriers and facilitators of public participation.

Two reviewers independently recorded data for each eligible article. Disagreements were resolved by discussion between the two reviewers or by assigning a third reviewer.

### Synthesis of results

The studies were grouped according to factors influencing public engagement in palliative care where we analysed the study one by one in a narrative way. Finally, a table was executed to summarize the barriers and facilitators of public engagement in PC.

## Results

**Fig. 1:**
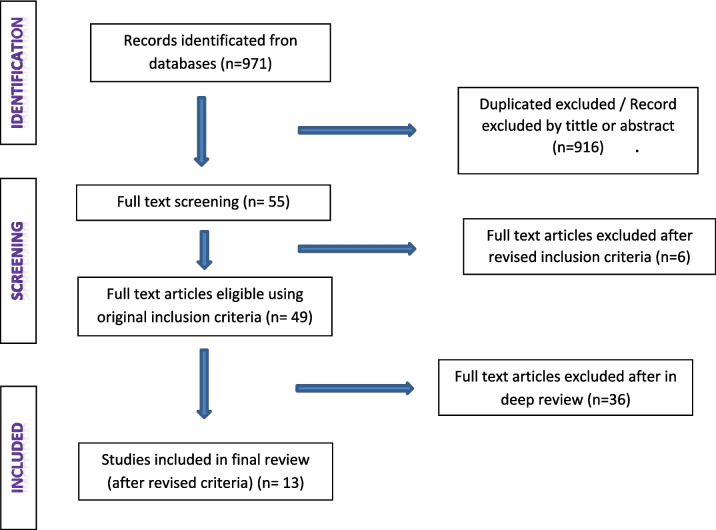
Flow-chart of study retrieval and selection process

A total of 971 articles were identified by databases searching. A total of 916 were excluded because they were duplicated or were excluded by title and abstract because they were considered not related with barriers and facilitators for public engagement. Fifty-five articles were taken to full-text screening but 6 were excluded because they did not match eligibility criteria and 49 were selected and two main authors reviewed in deep each of the full texts in relation to the inclusion criteria. Reviewers excluded 36 articles in a discussion session because they did not offer relevant information. We obtained 13 articles for the final analysis. Fig. 1 represents the selection process.

The characteristic of the selected articles is summarized in the Table 1. After analysis of the studies, they were broadly categorized into four categories to facilitate the description of the selected studies: 1. Public health and public engagement; 2. Community attitudes towards palliative care, death and preferences at the end of life; 3. Importance of volunteers in public engagement programs; 4. Compassionate communities. Finally, the factors that influenced public engagement in palliative care as barriers and facilitators for this engagement were identified and summarized in Table 2.

**Table 1:** Summary of the studies selected

Authors	Geographical Location	Design	Approach	Population	Key Findings
Sallnow L et al. (2016) [5]	Sweden, Southern Australia, Eastern Australia, Uganda, United Kingdom	Systematic review	Impact of community participation	Bereaved friends, family, caregivers of palliative, care patients, volunteers	Impact of community participation in care at the end of life. Important implications for policy makers, practitioners and researchers
Kumar S (2020) [17]	Southern Indian State of Kerala	Descriptive	Descriptive community participation	Community	Community participation is the only realistic model for achieving meaningful palliative care coverage
Sirianni G (2020) [20]	Canada	Descriptive	Descriptive	Community	Comprehensive, multilevel and multisystem approach to Public Health of palliative care
Collins A et al. (2020) [21]	Australia	Cohort study	Survey knowledge and attitude	Community	There are knowledge gaps about palliative care in the community. Public education programs can change attitudes towards Palliative, and community participation in them
Abba K et al. (2019) [22]	United Kingdom	Cohort study	Community intervention	Community	Well-designed community-based interventions can be successful in encouraging people to consider and discuss patients’ preferences at the end of life
Tieman J et al. (2018) [23]	Australia	Cohort study	MOOC	Community	Opinions and perceptions of the community around death can change with education. Indispensable for the development of resources and community participation
Pesut B et al. (2018) [24]	Canada	Pilot study	Community intervention	Volunteers	The volunteer program is a feasible option to foster a compassionate community approach to caring for an aging population
Warner G et al. (2021) [25]	Canada	Qualitative research	In Deep individuals and groupal interviews	Caregivers of palliative Volunteers	Barriers and Drivers to the implementation of volunteers program Nav-CARE
Loth CC et al. (2020) [26]	Africa	Descriptive	Key informant interviews	PC experts	Volunteers are beneficial for palliative care. Financing and long-term motivation are the main challenges
Librada-Flores S et al. (2020) [30]	Sweden, Australia, United Kingdom, Irlanda, Africa, Canada	Systematic review	Implementation, development and evaluation of CC	Caregivers, family, patients, volunteers, PC experts, community	Little evidence about implementation, development and evaluation models of CC for EoL
SECPAL (2020)	Spain, Colombia	Literature review	Situation review	Community	Community participation and methods of community participation in palliative care
Noonan K (2016) [46] and Leonard R et al. (2022) [22]	Australia	Descriptive	In Deep individuals and groupal interviews	Caregivers	Definition of Death literacy/ Development of an index to measure it
Grindrod A et al. (2018) [35]	Australia	Descriptive	In deep interviews, focus group and key informant	Caregivers, researchers and community	Identify key issue to build a CC at local level to provide a model to export (HELP program)

*MOOC* MAsive Open Online Course, *Nav-CARE* Navigation-Connecting, Accessing, Resourcing, Engaging, *CC* Compassionate Communities, *PC* Palliative Care

**Table 2:** Barriers and Facilitators for public engagement

**Subjects**		**BARRIERS**	**FACILITATORS**
**Patients / Families**		• Resistance to asking for help • Resistance to accepting help	Improving Compassionate Communities
**Health Care Professionals**		• Misperceptions of palliative care, knowledge gaps of palliative care, such as how to access and prognostic time left • Weakness in conversational skills in clinical practice • Not understanding Volunteer role in palliative care	•Training on EoL conversations, about values and preferences including knowledge and reflective approach •Help in understanding that Lessen isolation, providing information, giving emotional and social support, supporting HCP
**Public Health Care Systems**		• Fragmented care model • Non integration of health care systems with social welfare system • No palliative care Public health approach	•Improving a Shared Care Model of palliative care, integrating social welfare and promoting a Public Health Approach of palliative care •Improving Compassionate Communities
**Community**	**Unacceptance of death and dying talk in different social contexts**	• Resistance thinking about death and dying as a natural part of life • Social taboo of death due to social norms • Cultural, ethnical, religious, minorities differences can deter openness about death • Death and Dying are issues not addressed at schools, and teachers do not feel prepared to do so	•Improving a Shared Care Model of palliative care, integrating social welfare and promoting a Public Health Approach of palliative care •Improving Compassionate Communities
	**Emotional response to death and dying**	• Stress and fear thinking about death and dying (6) • Fear of upsetting others when approaching the subject of death and dying • Belief that others are unwilling to have conversations about death and dying	
	**Lack of interpersonal skills**	• Difficulty engaging others in conversations on death and dying • Concern over the skill to sensitively navigate conversations on death and dying	• Training including knowledge and reflection about Death and Dying, for example with "Last Aid courses" • Improving interpersonal skills training and working communication skills • Increasing awareness of different belief system, and inclusion of this in the communication skill training
	**Lack of knowledge on death and dying and of PC information**	• Misperceptions of palliative care, knowledge gaps of palliative such as how to access predict prognosis and predict time left • Online information can be confusing • Young adults are uninformed rather than misinformed	• Improving clarity on information and in training. Example dying learn MOOC • Need for Public education programs to provide targeted, accurate and consistent messages • Need to know more about palliative care, targeting layers of influence relating social networks, educational systems
	**And of PC information**	Young adults are uninformed rather than misinformed	Need to know more about palliativer care, targeting layers of influence relating social networks, educational systems
	**Difficulty involving citizens**	• Lack of time, continuity, components engagement • Inadequate preparation, conflicts of interest, political issues	Improving Compassionate Communities

*EoL* End of Life, *PC* Palliative Care, *MOOC* Masive Open Online Course, *HCP* Health care professional

### Public health and public engagement

Sallnow et al. [5] published in 2016 a Systematic Review on the impact of a new Public Health approach to end-of-life care. Three main themes emerged: making a difference to practice which describes the impact that public engagement can have on end-of-life experiences; individual learning and growth which describes the personal reflection, development and confidence that those involved embark on; and community capacity building, which refers to the impact of the work beyond the individuals involved, to the wider community where sustainable change can occur. The quantitative results were in line with the meta-ethnography and demonstrated that the involvement of communities can lead to improved outcomes for caregivers, such as decreased fatigue or isolation, increased size of caregiving networks, and that broader social networks can influence factors such as place of death and involvement of palliative care services. They argue that there is evidence of the impact of public engagement in end-of-life care, with important implications for policy makers, practitioners and researchers.

Kumar refers in his study to there being no general consensus on what is meant by community-based approaches to palliative care, yet public engagement is the only realistic model for achieving palliative care coverage for two-thirds of the world's terminally ill, especially as there is too much focus on the medical treatment of these patients [17]. Public engagement is described as a social process in which groups with shared needs living in a "defined geographical area" actively identify needs, make decisions and establish mechanisms to achieve solutions. For most communities at the local level, the level of involvement in palliative care programs will be at level 5 or lower in Pretty's typology [18, 19]. These projects usually have some form of community advisory board or committee, although they have limited decision-making capacity as these are dictated by the funding agencies. This makes it difficult to sustain the programs, as doing so, beyond funding, without the community having a real sense of involvement in decision-making is very difficult.

For the palliative care programs with higher levels of community participation, Suresh Kumar [17] detects some barrier focus on proactively building support networks within communities:

- Inadequate preparation: there are social and political challenges in setting up a community participation programme, including the diversity and inequalities in communities, knowing the social and political dynamics of the community, understanding and defining how communication between the facilitating team and the community will take place, knowing the values, beliefs and policies of external stakeholders (the facilitating organisation/institution/group, including funders and the global palliative care community, as well as the neighbourhood). This requires acquiring competencies through training programs.
- Different stages of the community development process: Different challenges within and outside the program structure that require different skill sets and approaches from facilitators.
- Organic nature of the community: It is difficult to decide who represents the community as it is not homogenous and is made up of different and sometimes conflicting interests. The main obstacles to developing and sustaining community partnerships are the voluntary nature of community participation, the enormity of the task and the natural conflict between community groups with different agendas and priorities. Community interventions are related to the social dynamics of the community, including power relations, economic conditions and vulnerabilities. Ongoing dialogue, learning and review mechanisms are necessary as communities evolve, adapting to changing interventions and contextual realities.
- Time constraints: Time constraints linked to grants often mean that there is insufficient time to adequately understand the community, engage multiple stakeholder groups with competing priorities and maintain project dynamism.
- Political issues and conflicts of interest: Differences in priorities and values between external experts/facilitators and the local community can lead to struggles for power and control of programs.
- Community participation policy: Empowerment of the local community is necessary. Empowerment is a multi-level concept that describes a process of social action to put people in control of their lives, their organisations and the lives of their communities. Through organisation and mobilisation, communities can achieve the social and political changes necessary to address their difficulties.
- Evaluation: It is not easy to track, document and monitor the process of community mobilisation. It is often not possible to do this using a standard research methodology (cause-effect) but rather a cascade methodology to measure impact.

Sirianni et al. [20] explored the possible difficulties in adopting a comprehensive approach in the community. They found "fragmented care" and difficult access resulting in instability and point to the need for policy involvement at all levels, state, provincial and local, along with the involvement of quasi-governmental agencies and non-profit organizations. They state that there is a need for the integration of a comprehensive, multi-level, multi-system approach to pallative care with well-resourced, palliative care-trained health care staff, public investment in palliative care services, financial support for patients/families, significant public literacy about the role of palliative care, and the implementation of a Compassionate Communities model at the national level. This approach involves a health promotion perspective on palliative care, harm reduction and early care for the patient and caregivers, with the involvement of health care providers, non-profit groups, faith-based groups, community organizations and public health providers to address both holistic patient care and public education about dying. They suggest that this comprehensive public health approach to palliative care can help with access, equity and cost.

### Community attitudes towards palliative care, death and preferences at the end of life

Collins et al. [21] described community understanding and attitudes towards palliative care and explore the characteristics significantly associated with favourable attitudes towards palliative care, understanding it as an enabler for public engagement. They found firstly, that public education programs can change attitudes towards, and thus community participation in, palliative care, and secondly, that there are knowledge gaps about palliative care in the community, which may limit access.

Abba et al. [22] conducted a follow-up study of a community intervention aimed at improving communication of end-of-life preferences and normalising death as a topic of conversation. Their intervention consisted of presentations and workshops aimed at community groups and people working in health and social care. Participants completed a survey in three phases: at baseline, after and three months after the intervention. There was a statistically significant association between increasing age group and having talked about end-of-life wishes. Most participants were already comfortable talking about the end of life.

Tieman et al. [23] state that ageing population, progressive diseases and end-of-life needs in hospitals and healthcare systems have a major impact on society. This has led to calls for public engagement with death and dying to encourage active participation in decision-making, community care and acceptance of death as a natural part of the life cycle. They carried out an intervention with the aim of allowing participants to discuss and learn in an open and supportive way about death-related issues, explore societal views and determine the effect that the online learning and discussions offered through the mediation had on participants' feelings and attitudes towards death and dying. The results showed that the mediation provided an opportunity to capture the views and perceptions of the community around death and dying, which they consider indispensable for the development of community resources and engagement.

Graham-Wisener et al. [6] consider that the main issue to involve the community in palliative care is the conversation about death and dying, which is aligned with the 'new public health approach' within palliative care. A key aspect is the normalisation of dying and the preparation of communities for the end of life. However, a significant proportion of adults report not feeling comfortable discussing death and dying with family and friends. This normalisation involves becoming 'death literate', which is defined as a set of knowledge and skills that enable end-of-life and death care options to be accessed, understood and acted upon.

### Importance of volunteers in public engagement programs

Three of the selected studies specifically discuss the importance of volunteers from the perspective of a public engagement approach to palliative care.

Pesut et al. [24] stated that volunteers providing supportive navigational services during the early phase of palliative are a feasible way to foster a compassionate community approach to caring for an ageing population. They piloted a compassionate community approach to early palliative care in several communities in Canada. They tested a capacity-building model in which volunteers and a nurse partnered to provide navigation support from the early palliative phase for adults living in the community, with the goal of improving quality of life by developing independence, engagement and community connections. Seven volunteers partnered with 18 clients. Throughout the trial year, volunteer navigators made home or phone visits every two to three weeks. Volunteers felt well prepared and found the role fulfilling and meaningful. Clients and their families felt that the service was very important to their care because the volunteer helped them to make the difficult experiences of ageing and advanced chronic illness more bearable. The most important benefits cited by clients were making good decisions for both the present and the future, having a substitute social safety net, supporting engagement with life, and ultimately transforming the experience of living with the disease.

Grace Warner et al. [25] conducted a qualitative study with the aim of using the Consolidated Framework for Implementation Research (CFIR) to explore barriers and facilitators to the implementation of a community-based volunteer program called Nav-CARE (Navigation-Connecting, Accessing, Resourcing, Engaging) for older adults with a serious health condition. They conducted qualitative individual and group interviews to examine the implementation of Nav-CARE in a Canadian community. Participants were individuals who conducted or managed NAv-CARE research, and stakeholders who provided services in the community. The results were organized into five themes that reflect participants' perceptions of Nav-CARE implementation:

1. Intra-organisational perceptions. Volunteers felt that they could provide information, new knowledge and a decrease in social isolation. Staff felt that the incorporation of volunteers could redistribute some of the workload, improve access to psychosocial support for the patient and their family. They expressed concern about boundary issues between the roles of volunteers and health professionals.

2.Public and health professionals' perceptions of palliative care. Stereotypical perceptions of palliative, misunderstandings in the community and health sector about who can access palliative, when and what palliative care involves. The public understands palliative care as synonymous with near death. Consequently, the programme associated with palliative care may be misinterpreted as appropriate only for people who will die in the near future. They see a need to "reframe" the perception of palliative care through community education and training. In addition, health professionals seemed to believe that palliative care should only be considered in the last months of life. Professionals recognised that it was their responsibility to educate the community about the need for early palliative care and the role of hospices. They emphasised that they should take a leadership role in promoting connections between hospices and community organisations to reduce fragmentation of care.

3. Partnerships and relationships between organisations. Participants expressed the need to educate and build relationships with community partners (e.g. pharmacists) with programmes (e.g. recreation centres) and with community groups that provide related services, such as a local caregiver support group. Building stronger partnerships between primary care and advanced palliative care teams with the idea of a 'shared care' model that is part of a palliative approach to care. Also, increased awareness in primary care practices of the need for end-of-life discussions. Engagement declined over time and health professionals did not fully understand the role of volunteers in patient care and that they lacked professional qualifications.

4.Factors at EU and national level. Development of resources, guidelines and training to help implement a palliative care approach in primary care. Creation of national legislation on medical assistance in dying (AMD). Lack of services made it difficult for primary care providers to consider referring patients to the programme.

5.Suggested changes to the programme. Several participants suggested several modifications to increase participation, such as facilitating paperwork, increasing training and internships, and increasing accessibility through Health Centres and churches. Loth et al. [26] published an exploratory study of palliative volunteers across Africa. They invited palliative care experts from 30 African countries to participate in an online survey consisting of 58 questions on: socio-demographics, activities, motivation and coordination of volunteers, and an assessment of recent developments in volunteering. Twenty-five respondents from 21 countries participated and the results showed a wide range of volunteering in palliative care. They identified volunteers as people between 30 and 50 years old, mainly non-professional women, motivated by altruism, a sense of civic engagement and personal benefit. They state that palliative care benefits from volunteers who take on a heavy workload and are close to patients and point to the main challenges of volunteer programs, problems of funding and motivation in the long term.

### Compassionate communities

The key elements for Compassionate Communities or Cities development models shared by several authors are social awareness and education programs on compassion and networks of care [27, 28], programs for training caregivers, neighbourhood network in palliative care [29] to provide home-based palliative care involving volunteers and the community and networks of care round people at the end-of-life initiatives with the implication of inner and outer networks, communities and service delivery organizations [2].

Silvia Librada-Flores et al. [30] evaluated models of Compassionate Communities and Cities (CCC) development at the end of life and their methods, processes and measures to enable evaluation of the intervention. They conducted a systematic review (from 2000 to 2018) in which they selected 31 articles, 17 descriptive studies, 4 intervention studies, 4 reviews and 6 qualitative studies. A total of 11 studies were on models of BCC (Behaviour change communication) development at the end of life, 15 studies were on the evaluation of BCC programs and 5 studies were on protocols for the development of BCC programs. This review reflects the growing development of CCC that has been launched. The model described by Kellehear A [1, 31] has helped to orient these initiatives towards the elements that characterise the development of a Compassionate City. Published recommendations and coalitions on BCC development also reflect the empowerment of this movement from public health and palliative care policy in an integrative health-social-community care model.

Although this review provides interesting information on recommendations and an approach to models, methods and evaluation systems for BCC, the quality of this evidence is low or very low. Most are descriptive or proposals for future interventions based on literature reviews. There are no studies with representative samples and/or randomised methodology to provide more accurate information on the benefits of these interventions. The evolution of some of these programs, whether they are pilot programs or still ongoing, is unknown. No studies have been identified that demonstrate the opportunities or difficulties in implementing compassionate cities and communities’ projects. Furthermore, a comparison between the different initiatives developed cannot be made due to the method used and the absence of quantitative results. Despite all these limitations, these results serve to guide models on the benefits of these programs and further research is needed to clarify and improve our knowledge. As this is an emerging movement, the described experiences should also go in this direction to guide other cities and organisations [30].

The Spanish Society of palliative Care (SECPAL) published in 2020 a Monograph on Compassionate Communities at the End of Life, where more than 30 authors contribute their knowledge and experiences to create accompanying networks in schools, universities, neighbourhoods and other social organizations in order to provide support to patients with a life-limiting illness and their families [32].

The substantial difference of a Compassionate Communities Program from isolated public engagement initiatives is the articulation of an Integrated Health, Social and Community Care Model for advanced illness and end of life under an organizational system that manages, coordinates and evaluates it and with a methodology for implementation, monitoring and evaluation [33].

For the development of this Model, it is necessary to have [34]: Promoter leadership in technical, professional and economic terms; Definition of an area of coverage; Annual work plan, with a program of actions and specific objectives; Institutional collaboration; Generation of community intervention structures; Community intervention protocols and design of tools; Arrangement and activation of a network of promoting agents with material and human resources; Design of an evaluation system; Communication and dissemination of the program; and Publication of tools and results.

The aim is to raise awareness, train and intervene in the creation of networks (internal and external) and in the action of these networks for people with advanced illnesses and at the end of their lives; managing to evaluate their impact and results in terms of patient and family satisfaction, professional satisfaction, impact on health (improvement of quality of life, reduction of carer overload, reduction of depression and anxiety and increase in the average number of carers). Of this way avoiding duplication and inefficiencies in the use of available resources in the specific geographical area in which they are developed, and generating new community structures (such as community connectors, the community promoter, the dynamic commission and the socio-health commission) [34]. Several of the Compassionate Cities that are currently underway describe the barriers and facilitators to their development in the Compassionate Communities at the End-of-Life monograph [30, 32, 35]. These barriers and facilitators are collected in the Table 3.

**Table 3:** Barriers and Facilitators for Compassionate Communities (CC)

Compasionate Communities	Barriers	Facilitators
Getxo Contigo	• External Team • Little institutional relationship in the beginning • Transversability of the project that sometimes made key agents understand that it was not their area • Taboo topic in our society • Youth of the promoting entity	• Public engagement from the beginning • Methodology used • Latent needs in the community
Pamplona Contigo	• Difficulty in specifying the participation of some entities due to lack of sufficient own personnel • Difficulty in networking with some entities	• Good willingness of the entities contacted to work together • Have a public network of health and social services
Programa Vic. Ciudad Cuidadora	• Openness to different groups and cultures • Dissemination in the media • Promotion of internal actions in organizations • Systematic evaluation of results	• Shared leadership local administration and professorship • Effective coordination • Participation of various entities • Defined objectives
Sevilla Contigo. Ciudad Compasiva	• Access of community promoter to people with advanced disease and/or end of life is mediated by the referral of social-health professionals and by the community, but perhaps they are not directly visible on the radars of the social-health systems • Limited intervention time • Initial distrust of the people potentially benefiting from an innovative and unknown program • Difficulty accepting help from other members of the community • Fear of social stigma	• Support from key entities such as the Seville City Council, community social services, the Andalusian Health Service, health centers and hospitals • Involvement of the community itself in the direct execution of the project, reflecting on the importance of community work in caring for people with chronic, advanced and/or end-of-life illnesses • The commitment and involvement of the socio-sanitary professionals • The participation of key agents in the project
Victoria-Gasteiz Ciudad Compasiva: Vivir con Voz Propia	NA	• Development of various community programs and actions (Space for reflection and dialogue, Sensitization about the elderly and the end of life in educational centers, Young People with their Own Voz, Adult Volunteer Group, Death Café, Participation in socio-community activities in Victoria-Gasteiz • Collaboration of international experts and research
Zarautz Pueblo Cuidador	NA	• Cohesion within the promoter group • Municipal involvement • Good harmony with other municipal associations and with other compassionate communities
Cali, Ciudad Compasiva. Red Unidos por la Compasión	• Rotation of members, sometimes according to the needs of the entities • Low willingness of government entities to join the project • Heavy workloads of the members	• The human resources that has managed to develop trust, motivation and commitment processes • Teamwork of members with different types of training that enrich dialogue • Commitment and personal motivation, beyond the institutions they represent • Awareness of the need to develop systematization activities and indicators that allow monitoring and assess the relevance of the resources used
Medellín: Red Compasiva, Transformación Social para el Cuidado	• Limitations of human, financial and time resources • Dependence on the voluntariness of organizations to make contributions to the network	• The need felt to structure support networks and articulate the efforts of multiple and diverse organizations in the city • The existence of the public policy for caregivers and the political will • The scientific, academic and financial contribution of the Pontifical Bolivarian University (UPB) • The population's interest in caring for others and the value given to compassion
Healthy End Of Life Project (HELP)	• Identify, engage and support local people • Place-based approaches incorporate end-of-life support into existing social and community structures and settings to meet local need • Creative community initiatives based on local strengths and interests • Local solutions through creative collaborations between community health and social organizations and individuals • Coordinate local responses that aim to overcome structural barriers, change community culture and improve individual healthy end of life planning	• The reluctance of carers to accept help when it was offered by family, friends and neighbours • Ask for assistance from their existing support networks • Repeated offers with repeated refusals, are likely to feel intruding, assume assistance is not required, or feel personally excluded • Ways of identifying local residents in need of support • Seeing dying, death and bereavement as a private matter undermined initiatives to improve informal social care at the end of life • Invisibility of end of life matters in the community

*NA* Not applicable, *CC* Compasionate Communities

Public Health Palliative Care International (PHPCI) [8] recommend that for a public engagement and involvement a Compassionate City should develop and support 13 key social changes and activities. PHPCI [43] recommends that, to achieve public engagement and involvement, a Compassionate City should develop and support 13 key social changes and activities. Both schools and workplaces should have guidelines, reviewed annually, on bereavement, death, bereavement and care. Churches and houses of worship should have at least one group dedicated to end-of-life care support. Hospices and nursing homes should have community development programmes that involve citizens in end-of-life care activities and programs. They should also involve museums and art galleries, hold memorial parades, promote compassionate communities programmes to engage local neighbourhoods or streets in direct care activities for their local residents living with health, aging, caregiving and bereavement crises. Create incentives to celebrate and highlight the most creative and compassionate organisations, events and individuals. Also publicly showcase through media, social media, public events by policy makers, compassionate initiatives undertaken or underway that help raise awareness about ageing, death, loss or caregiving. Establish social and political alliances that take into account the diversity of populations within the same city, neighbourhood or street. Finally, to encourage and invite evidence that institutions and organisations are working together to promote and support the development of a common understanding of ageing, death, bereavement and caregiving.

## Discussion

This scoping review has identified public health factors, with implications for policy makers, professionals, researchers, organizations and the community in different settings: caregiving institutions (hospitals, nursing homes, health centres), capable of detecting people in need of palliative care, volunteer organizations (associations, NGOs and religious organizations) that offer accompaniment and participation resources, and political entities (state, provincial, local) that make it possible to optimize the resources already available to the community.

The literature identifies a number of barriers, including lack of knowledge of the death system, fear or distress associated with thinking about death and dying, and difficulty in engaging in conversations about death with others or fear of upsetting others. Several levels of barriers can be identified, such as social perception and practice (death as a social 'taboo'), lack of opportunities (perceived lack of family and friends to talk about it with) and support and personal emotions and values (concern about causing distress [6, 36, 37]. In relation to facilitators, we can observe improved acceptance of death as part of life or the use of a public health approach to engage people [6, 37].

Social norms can place limits on opportunities to talk about death, and it is believed that these conversations should only take place within families and in particular circumstances. This is an important constraint when people believe that their family members are unwilling to talk about death. It is unclear whether this is related to death as a psychological taboo or rather suggests shame in talking about death. Another concern is the handling of emotions during these conversations with family and friends [6]. This suggests that there is value in raising awareness and accessibility of safe spaces, such as Death Cafes, to discuss death and dying with members of the wider community. The aim of Death Cafes includes helping people to express emotions they do not feel able to express elsewhere. However, like all initiatives, they have their limitations, for example, there are no formal evaluations of these initiatives to compare and assess their real impact. Furthermore, these initiatives, for now, seem to have a very specific audience, middle-aged women working in the health sector, so it is necessary to consider how these initiatives can be optimised to involve "hidden audiences", such as young people and men [38, 39].

The perception that others are unwilling to talk about death relates to a key facilitator of the importance of normalising the discussion of death and dying. In this context, educational settings are perceived as an opportunity to engage children and young adults, with a life course approach to talking about death and dying (respondents equated this with 'sex education'). Although research on children's perceptions of death is scarce, a model of 'death ambivalence' is offered in which children both avoid death and cope with it [40]. The avoidance of death was largely the result of the social domains of which the children were a part (family and education), in addition to broader cultural norms about what it means to be a child. There is an openness and desire for information and discussion about death on the part of children, and recent research in Spain indicates that parents favour the inclusion of death education in their children's education [41]. Recent research in Northern Ireland also suggests that there is value in integrating death education into the university education of young adults, where a high level of awareness but lack of knowledge around palliative care is reported [42].

Concern about the interpersonal communication skills of both self and others in talking about death and dying. The need to foster individual responsibility for initiating these conversations was identified, but the main focus was on equipping those concerned with the 'tools' to do so. Particular attention has been given to the development of evidence-based, peer-led advance care planning (ACP) facilitator training programmes. This has facilitated discussions about ACP and the completion of advance care directives, as well as the provision of ACP education, training and support. Most of this training focuses on training volunteers to facilitate ACP discussions with older adults or clinical populations [43]; however, there is an evidence base that supports these support programmes being done by people in the community who facilitate discussions with people close to them [44].

Concern that talking about death offends or distresses people with strong spiritual or religious beliefs. An increasingly multicultural society and adults who identify as non-religious, resulting in communities that are increasingly diverse in relation to spiritual or religious beliefs. Weisener et al.'. showed that increasing awareness of different belief systems made things easier, so it seems an important component of interpersonal communication skills training for contemporary society [6].

One of the facilitators to help to the community to care is the diagnosis of how communities come together to care, this is the death literacy [45, 46]. Death literacy is defined as “a set of knowledge and skills that make it possible to understand and act upon end-of-life and death care options” [46]. Such skills strengthen individual and community capacity to act and care for each other through the experiences of dying, death, loss and bereavement. The four facets of death literacy are knowledge, skills, experiential learning and social action. People involved in care networks report increased knowledge of services, health policies, medical procedures and end-of-life planning. Also important are acquiring skills in self-care, in having conversations about death and the dying process, in negotiating with health and other service providers, and in caring for and disposing of the body. Experiential learning showed how attitudes and beliefs were transformed by the experience of caring, leading to greater acceptance of death as part of life, the normalisation of informal care and a deeper appreciation of the privilege of caring and the importance of involving others. In addition, it was observed that those with knowledge and skills derived from their caring experience shared these with their networks. They recognised that they were better equipped for end of life care and felt able to re-engage care networks when necessary [45].

One of the basic keys to raising awareness and public engagement is to know what society is like and what it can offer, identifying and bringing together the values, resources and experience it already has, its "assets" [9]. For this issue the diagnosis of death literacy would be very helpful to know how the communities come together to care. Death literacy is aligned with public health approaches to palliative care [4, 27].

The community is already compassionate, but needs to channel that potential. Compassionate public health "Third wave public health" incorporates the experiences of death and loss into our health formulations and includes the idea of compassion in our health policies and practices.

The challenge is to involve the community through awareness-raising actions that provide citizens with tools to know how to care for and accompany people in need and to articulate the help of those who make up these networks. This task can be channelled through health professionals by connecting the important assistance part (recognition of the person in need) with community resources. In addition, there is a need to systematically evaluate the process and the long-term results of these initiatives [32].

This strategy is in line with the public health approach in palliative care and allows catalysing community participation through four fundamental steps: identification and participation of social agents, associations and public entities: meeting, so that the community and organizations can work together; a clear action plan, which reflects the needs of the community with well-defined objectives; implementation and evaluation of the effectiveness of the program [10].

In order for the community to be actively involved, the key points are [10]:

1. Information, raising awareness of the importance of an integrated community in palliative care to the extent that the various agents of the community become aware of this and of the repercussions it will have in their context, making their participation greater.
2. Dissemination, messages, campaigns, dissemination events, facilities to join, programs put in place, essential elements to approach a living community model in which everyone can find viability in the help they can offer.
3. Education, training through educational programs. Educational centres are a basic element in the generation of knowledge, but also in the involvement with the community through training in values, such as solidarity, respect and care for the most vulnerable, especially at an early age.
4. Leadership: the existence of a coordinator, and the identification and participation of all social agents, associations and public entities, which have influence in the target community and allow the detection of needs.
5. Viability and Sustainability: one of the most important challenges is to raise awareness among the citizens of the future so that they assume and understand the social value of care, which is why it is necessary to start raising awareness in schools. This also has implications for politicians and managers, who must be willing to participate, trust and finance these initiatives, allowing communities to develop and customize these initiatives within their context. There is also a challenge at the research level as there is a need for evaluation processes and the identification of outcome measures that can assist us in assessing the effectiveness and improvement of these initiatives. Finally, the identification of other awareness-raising initiatives allows for broader networking and learning from each other.

To diagnose and assess the progress of public engagement, the Death Literacy Index (DLI) [45] is a measure that can provide policy makers with strategies to improve wellbeing in the end of life through efficient and effective use of resources. Furthermore, the DLI underlines the idea that these resources can be both formal and informal, and that the capacity of the community to provide care must be taken into account in policy and practice at the end of life. The DLI has shown sensitivity to measuring changes that occur as a result of programmes that improve a community's capacity to provide end of life care [45].

In recent years, major sociodemographic changes have taken place around the world, forcing us to rethink the approach and organization of health services to adapt to them. The ageing, dependency and loneliness of the population, technological development, changes in the role of the patient and the current socio-economic situation, among others, are elements that mark this new scenario. An example of movement about it is the "Compassionate communities: for a global community united by the vocation of caring" led by PHPCI arises [9].

Palliative care began with, and continues to emphasize, tertiary level interventions that emphasize inpatient facilities and specialized service providers. A primary health care approach is evident in some parts of the world with extensive use of general practitioners or community nurses who provide initial assessment and share responsibilities with specialists in providing appropriate interventions. A community-based health approach is the least developed in palliative care services. However, it is the approach that has the greatest potential to improve quality of life and sense of well-being for the greatest number of people who are ill and in health, in death and bereavement, and in all experiences of mutual care [5].

The major limitation of this review is that a standardized critical evaluation of each article was not performed due to the scarcity of the literature and variations in the methodology and quality of the articles reviewed. Most of the articles initially analysed the barriers and facilitators of PCs for the community, the latter being an end and not a resource in itself. Furthermore, here is little literature aimed at studying the factors that favour or hinder community participation in palliative care [47].

## Conclusions

The current interpretation of palliatuve care as clinical end-of-life care, have implicit that patient and family care must preserve the dignity of all people involved, although this does not mean that it encompasses public engagement in end-of-life care. For that reason, societal awareness must be a facilitated process focused on community assets to catalyse public engagement efforts across sectors at the community level. National policy initiatives and regional system support provide legitimacy and focus, and leadership from them is essential for funding.

The challenge is to involve the community through awareness-raising actions that provide citizens with the tools to know how to care for and accompany people in need and to articulate the help of those who make up these networks. This task can be channelled through health professionals by connecting the clinical assistance part (recognition of the person in need) with the resources of the community. In addition, there is a need to systematically evaluate the process and the long-term results of these initiatives.

It is the real commitment of the people and organizations working in the community who possess the skills and experiences necessary to bring about change.

## Data Availability

Data used in this manuscript consist of published articles which cannot be shared by the authors for copyright reasons but are available through subscription to the relevant journals/databases.
